# COVID-19 Vaccine Willingness and Education Level: A Multinational Cross-Sectional Analysis

**DOI:** 10.7759/cureus.85544

**Published:** 2025-06-07

**Authors:** Samantha R Karp, Youry B Jean, Opeoluwa A Ashiru, Shahid B Rangrej

**Affiliations:** 1 Medical School, Saint James School of Medicine, Arnos Vale, VCT; 2 Anatomy/Research, Saint James School of Medicine, Arnos Vale, VCT

**Keywords:** coronavirus covid-19, covid 19, covid 19 vaccine, level of education, public health and safety, socio demographic factors, vaccination uptake, vaccine hesitancy, vaccine refusal, vaccine willingness

## Abstract

Purpose

This study aims to examine the relationship between individuals' education levels and their willingness to receive the COVID-19 vaccine. Understanding vaccine willingness and its influences allows for the identification of vulnerable communities in the face of global viral pandemics such as COVID-19. By identifying vulnerable populations, such as individuals who have restricted access to education, public health officials can create more effective targeted interventions to increase vaccine willingness and uptake.

Methodology

Surveys were administered online and in person, targeting adults aged 18 years or older, and were designed to achieve broad geographical reach. Participants self-reported their highest level of education and rated statements related to COVID-19 vaccine willingness, perceived importance, and understanding. Data were analyzed using Microsoft Excel for Mac (version 16.70 23021201) (Microsoft® Corp., Redmond, WA).

Results

Results from our study demonstrate that individuals with higher education levels were significantly associated with greater vaccine willingness (B = -0.99, p < .001); since reverse scoring was utilized, lower willingness scores reflected greater willingness. The regression model accounted for 36.4% of the variance in willingness (R² = 0.364), suggesting a moderate to strong relationship within our sample.

Conclusion

As vaccine hesitancy remains a global challenge, our findings highlight education level as a potential strategy for addressing vaccine willingness across multiple countries within our sample. Having a well-rounded understanding of the key factors that influence vaccine willingness is significant for informing efficient public health policies.

## Introduction

The global effort to control the COVID-19 pandemic was significantly hindered by vaccine unwillingness and uncertainty worldwide. An individual’s unwillingness to be vaccinated may be influenced by various factors, including education level. This survey-based study aims to evaluate the relationship between education level and vaccine hesitancy within our multinational sample. The study predominantly includes responses from Canada, the United States of America, and Saint Vincent and the Grenadines. Participants were asked to rate their education level and score their willingness to be vaccinated with the COVID-19 vaccine, the importance of being vaccinated with the COVID-19 vaccine, and their understanding of vaccination programs. Understanding vaccine hesitancy and its influences is crucial for health authorities to implement effective immunization campaigns, which are essential for reducing the burden on healthcare systems, protecting vulnerable populations, and ultimately controlling the spread of a virus.

## Materials and methods

Surveys were distributed both in person and online via social media platforms (Facebook (Meta Platforms, Inc., Menlo Park, CA), LinkedIn ((Meta Platforms, Inc., Menlo Park, CA)), Instagram (Meta Platforms, Inc., Menlo Park, CA)), targeting adults aged 18 years and older (Appendices). In-person data collection employed convenience sampling at publicly accessible locations such as apartment complexes and grocery stores. Online participants were recruited through a publicly shareable web link generated by SurveyMonkey (Momentive Global Inc., San Mateo, CA), which was disseminated across social media channels. Online responses were collected via SurveyMonkey, ensuring participant anonymity and data security. Incomplete responses and surveys missing key variables were excluded from analysis, resulting in a final sample size of 458 participants. The survey was available online from February 3rd to March 11th, 2023.

All statistical analyses, including simple linear regression, Pearson’s correlation coefficients, and chi-square tests, were conducted using Microsoft Excel for Mac (version 16.70 23021201) (Microsoft® Corp., Redmond, WA). Statistical significance was set at p < 0.05. Multivariate regression was not applied, as this exploratory study primarily aimed to examine the direct relationship between education level and vaccine attitudes. Microsoft Excel was selected for its accessibility and suitability for the dataset size. The response rate for the study was 90%. The complete survey instrument is included as a supplementary file in the article appendix. Although convenience sampling was used, participant consent was obtained, and privacy was maintained throughout the study.

Surveys were open internationally and predominantly included responses from three primary countries: Canada (n = 187), the United States of America (n = 190), and Saint Vincent and the Grenadines (n = 57). In addition to the three primary countries, the remaining responses were acquired from Germany, India, Jamaica, Mauritius, Mexico, Nigeria, Portugal, Romania, the United Kingdom of Great Britain and Northern Ireland, and Zimbabwe, resulting in a total sample size of 458 participants. Demographic data (age, gender, country of residence) were also collected.

A simple linear regression analysis was performed to examine whether reported education level predicted COVID-19 vaccine willingness, with willingness scores reverse-coded so that lower values indicated greater willingness. Pearson’s correlation coefficients were calculated to assess relationships between education level and vaccine willingness, importance, and understanding. The chi-square test of independence evaluated differences in vaccine willingness across education-level groups. 

Participants were asked a single-item measure to assess COVID-19 vaccine willingness: “Rate your willingness to have received the COVID-19 vaccine” rated on a five-point scale (1 = very willing; 2 = somewhat willing; 3 = not so willing; 4 = not at all willing; 5 = not at all willing and did not get vaccinated). Education level was assessed on a four-point scale (1 = some high school; 2 = completed high school; 3 = some college or university; 4 = completed college or university). Respondents who selected "some high school" were interpreted as individuals who had not finished high school and were no longer enrolled, consistent with the age requirement of 18 years and older for study participation.

Understanding of vaccine program rationale, as well as the perceived importance of vaccine programs, was also assessed in this study. Participants were asked to rate the importance of receiving the COVID-19 vaccine by responding to the statement: “Receiving the COVID-19 vaccine is important for protecting myself and my community” on a five-point Likert scale (1 = strongly agree; 2 = agree; 3 = neither agree nor disagree; 4 = disagree; 5 = strongly disagree). Similarly, subjects rated their understanding of vaccine rationale using the statement: “I understand how vaccines help reduce the spread of disease in communities and help establish herd immunity” on the same five-point Likert scale.

## Results

Among 458 participants, the median age range was from 25 to 34 years; most were female (64.05%), and of the 458 participants, 314 (68.41%) reported having completed college or university at the time of the survey. Our study utilized a simple linear regression to examine whether our sample’s reported level of education predicted willingness. Willingness was reverse-scored such that lower values indicated greater willingness.

As shown in Table 1, the regression coefficient for education level was negative and statistically significant (B = -0.99, SE = 0.06, t = -16.18, p < .001, 95% CI (-1.11, -0.87)). The overall correlation between the level of education and vaccine willingness was found to be R = 0.60 (p < 0.001). This indicates that individuals with higher levels of education tend to have lower willingness scores. Given the reverse scoring, this suggests that higher education is associated with greater willingness.

**Table 1:** Linear Regression Analysis of Education Level Predicting Willingness

Predictor	B (Unstandardized Coefficient)	Standard Error	t-Value	p-Value	95% CI (Lower)	95% CI (Upper)
Intercept	5.49	0.22	25.16	<0.001	5.06	5.92
Education Level	-0.99	0.06	-16.18	<0.001	-1.11	-0.87

The overall regression model was statistically significant, F(1, 457) = 261.81, p < .001, and accounted for approximately 36.4% of the variance in willingness scores (R² = 0.364, Adjusted R² = 0.363) as summarized in Table 2. These findings suggest a moderate to strong relationship between education and willingness in our sample. Chi-square analysis reveals that in our sample, the measure of COVID-19 vaccine willingness was found not to be distributed equally between education-level groups (χ^2^ = 206.59; p < 0.001; degrees of freedom = 12).

**Table 2:** Model Summary df, degrees of freedom

Statistic	Value
R	0.604
R²	0.364
Adjusted R²	0.363
Standard Error of Estimate	1.178
Observations (N)	459
F-statistic (df = 1, 457)	261.81
Model Significance (p)	<0.001

COVID-19 vaccine willingness

**Figure 1:**
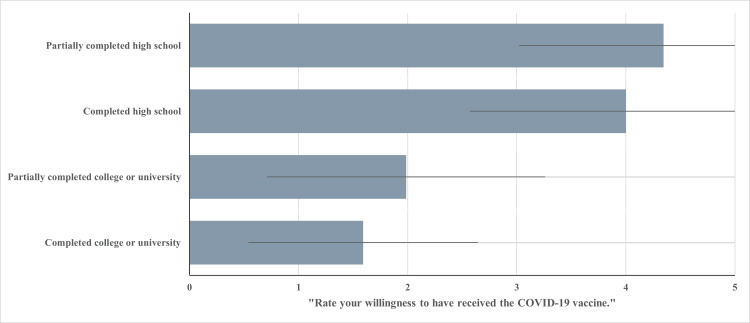
Education Level and Mean Willingness Score Level of education achieved vs. mean score of participants when asked to “Rate your willingness to have received the COVID-19 vaccine” rated on a five-point scale (1 = very willing; 2 = somewhat willing; 3 = not so willing; 4 = not at all willing; 5 = not at all willing and did not get vaccinated).

The extent to which participants were willing to receive the COVID-19 vaccine was determined using a single-item measure, with scores closer to one indicating high willingness and scores closer to five indicating low willingness. Figure 1 visualizes the results for the relationship between reported education level and the level of reported willingness to receive the COVID-19 vaccine. The mean score for willingness to receive the COVID-19 vaccine among those who self-reported to have partially completed high school (n = 26) was found to be 4.35 (SD = 1.32). For those who completed high school (n = 51), a similar mean score for willingness was reported at 4.00 (SD = 1.43). For those who completed some college or university (n = 68), the mean score for willingness to receive the COVID-19 vaccine was 1.99 (SD = 1.28), and for participants who completed college or university (n = 314) were found to have the lower mean score for COVID-19 vaccine willingness with a score of 1.59 (SD = 1.05).

Subgroup analysis revealed that the highest ratings of vaccine willingness (M = 1.42; SD = 0.91) were found among men who completed college or university (n = 101), while men who have partially completed high school (n = 17) are shown to have the highest mean score on this measure, indicating low ratings of vaccine willingness (M = 4.71; SD = 0.99).

COVID-19 vaccine importance

Education level was found to be moderately correlated with participants’ ratings of vaccine importance (R = 0.52; p < 0.001). Participants who have partially completed high school have an average mean score of 4.0 (SD = 1.20), indicating disagreement with the statement “Receiving the COVID-19 vaccine is important for protecting myself and my community”. Individuals who have completed high school have a similar score, indicating slight disagreement with the statement of vaccine importance (M = 3.41; SD = 1.27).

**Figure 2:**
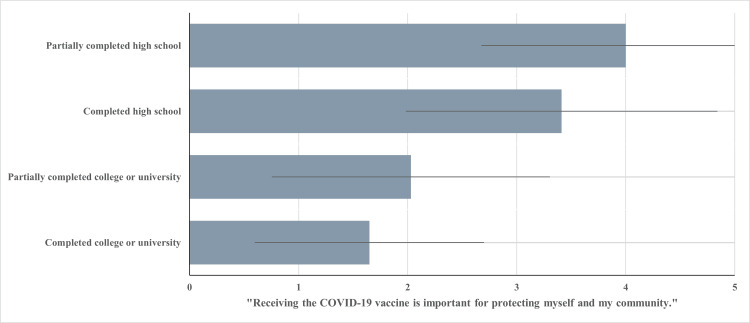
Education Level and Mean Importance Score Level of education achieved vs. mean score of participants when asked to rate the following statement: “Receiving the COVID-19 vaccine is important for protecting myself and my community”, on a five-point scale (1 = strongly agree; 2 = agree; 3 = neither agree nor disagree; 4 = disagree; 5 = strongly disagree).

Participants who have partially completed college or university have reported mean scores of 2.02 (SD = 1.20), implying agreement with the statement of vaccine importance. Participants who have completed their college or university program have shown to have a mean score of 1.65 (SD = 1.03), indicating a high level of agreement with the statement of importance for COVID-19 vaccines. Figure 2 visualizes the results for the relationship between reported education level and the level of reported COVID-19 vaccine importance.

COVID-19 vaccine understanding

Reported data for participants’ understanding of how vaccine programs function to protect individuals and communities was found to be moderately correlated with education level (R = 0.64; p < 0.001). Individuals who partially completed high school (n = 26) demonstrated a low understanding of the rationale of COVID-19 vaccination programs with an average response of “disagree” when asked to rate their opinion of the statement: “I understand how vaccines help reduce the spread of disease in communities and help establish herd immunity” (M = 4.0; SD = 1.10). Individuals who have completed high school (n = 51) had a mean score of 3.15 (SD = 1.14).

**Figure 3:**
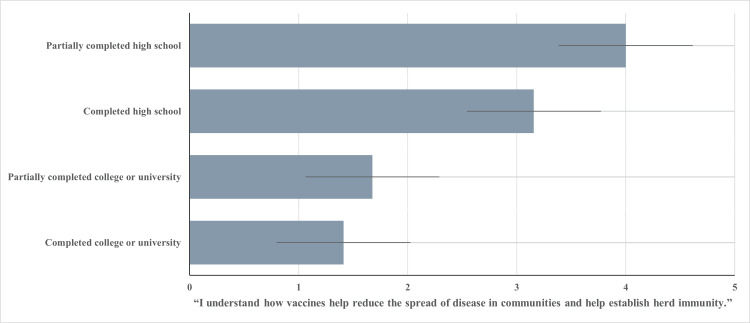
Education Level and Mean Understanding Score Level of education achieved vs. mean score of participants when asked to rate the following statement: “I understand how vaccines help reduce the spread of disease in communities and help establish herd immunity” (1 = strongly agree; 2 = agree; 3 = neither agree nor disagree; 4 = disagree; 5 = strongly disagree).

Those who reported partially completing a college or university program had a mean score of 1.68, revealing a good understanding of vaccination programs (SD = 0.74). The highest level of understanding based on ratings of the statement was reported among participants who have completed their college or university programs (M = 1.41; SD = 0.80). Figure 3 exhibits the results for the relationship between reported education level and the level of reported COVID-19 vaccine understanding for this study.

Relatedly, data reported on understanding of COVID-19 vaccination programs strongly correlated with the level of vaccine willingness among participants (R = 0.80; p < 0.001).

## Discussion

This study aimed to explore whether an individual’s education level relates to their willingness to receive the COVID-19 vaccine. The results revealed a statistically significant relationship between higher levels of education and greater willingness to be vaccinated. Specifically, simple linear regression demonstrated that education level accounted for approximately 36.4% of the variance in vaccine willingness, a moderate-to-strong association within our sample. These findings add to growing evidence suggesting that education is associated with differences in public health behavior, particularly in the context of vaccine willingness.

Several researchers emphasize that vaccine hesitancy is multifactorial, with education referenced as a potentially modifiable determinant [1,2]. Existing literature has also previously demonstrated this relationship in adolescents, suggesting that the education-willingness link emerges early in life [3]. Similarly, global surveys have identified education as a consistent determinant of vaccine acceptance across international populations [4,5].

One reasonable explanation for this trend is that individuals with higher educational attainment tend to possess greater health literacy, enabling better comprehension of scientific and public health information [6]. This improved understanding may result in greater trust in the medical system and a higher likelihood of following health guidelines. A previous systematic review also concluded that confidence and receptivity toward COVID-19 vaccines are closely tied to education level, further supporting the role of education in shaping vaccine attitudes [7]. Research has additionally highlighted how mistrust in biomedical research correlates with vaccine hesitancy, particularly in countries like Italy, where public skepticism has historically been high [8]. Moreover, individuals with higher education levels are generally less susceptible to health misinformation, which has played a major role in vaccine hesitancy.

The impact of misinformation on vaccine decisions is well-documented. Previous research has found that exposure to misinformation significantly decreased willingness to vaccinate in both the UK and the US [9]. Other studies have demonstrated that misinformation on social media platforms contributed directly to vaccine hesitancy [10]. These findings underscore the potential value of designing public health interventions that are not only educational but also aim to reduce the impact of false or misleading information, particularly among populations with lower educational attainment.

Our findings may offer preliminary insight into how targeted public health messaging could be structured. Campaigns aiming to increase vaccine uptake should prioritize clear, accessible communication and consider tailoring strategies for individuals with lower levels of formal education. These efforts should involve improving access to credible information and fostering trust in public health institutions. It is important that governing health authorities identify vulnerable populations that are more susceptible to misleading or inaccurate information and subsequently increase their access to accurate, comprehensible health information.

To further contextualize these findings, the Health Belief Model (HBM) offers a useful theoretical framework. The HBM suggests that individuals' health behaviors, such as vaccine uptake, are influenced by their perceptions of susceptibility to disease, the severity of the disease, the benefits of taking action, and the barriers to action [11]. In our study, higher educational attainment may correlate with increased health literacy, potentially enhancing individuals' perceived benefits of vaccination and reducing perceived barriers related to misinformation or mistrust. Moreover, participants who reported a greater understanding of vaccine rationale may reflect higher self-efficacy, another key construct of the HBM. By interpreting our results through this behavioral lens, the relationship between education and vaccine willingness can be better understood not only as a demographic association but also as a reflection of cognitive and psychological processes that influence public health behavior.

A key strength of this study is the use of online surveys distributed across multiple social media platforms, which facilitated participation from several countries and demographic groups. This approach helped mitigate some country-specific biases, such as political or cultural influences on vaccine willingness. The use of both online and in-person formats further enhanced accessibility, reaching participants with varying levels of digital literacy or internet access. The study also assessed multiple dimensions of vaccine attitudes, including willingness, perceived importance, and understanding, offering a broader perspective on factors influencing vaccine acceptance. These strengths provide valuable exploratory insights that may inform the design of targeted, accessible public health interventions.

Despite these strengths, several limitations should be noted. This study focused primarily on education level as the key predictor, while other potentially relevant factors such as income, political affiliation, and healthcare access were not included. This narrows the theoretical scope and highlights the need for future research to investigate a wider range of predictors using validated multi-item instruments. Furthermore, single-item measures were used to assess vaccine willingness, perceived importance, and understanding. Although chosen for clarity and ease of interpretation, these measures may not fully capture the complexity of these interrelated constructs. Employing multi-item, validated scales in future studies would enhance construct validity and allow for more nuanced analysis.

Additionally, the cross-sectional design of this study provides a snapshot of associations at a single point in time, which limits the ability to infer causal relationships. Although the sample includes participants from multiple countries, the findings primarily reflect correlations rather than cause-and-effect. Therefore, while the results offer useful insights, they should be interpreted with some caution when considering implications for public health interventions across different settings. Future longitudinal or experimental research would be valuable to better understand causal pathways and to develop tailored, context-specific strategies to enhance vaccine uptake.

The sample for this study was also self-selected and may not be representative of each country’s broader population. Recruitment relied on convenience sampling, both online and in-person, which may introduce selection bias. This may have skewed the sample toward individuals who are more digitally engaged or already interested in public health issues. Social media-based recruitment may also have disproportionately attracted younger, more educated individuals. These factors may limit the generalizability of the findings. Future research should consider using stratified or random sampling techniques, as well as targeted recruitment strategies, to improve representation across demographics and reduce platform-related biases. Additionally, future research could also consider examining the influence of educational discipline to better understand how specific knowledge domains shape attitudes toward public health interventions, this study focused on the level of education rather than the specific type or field of education received. It is possible that individuals trained in health-related or scientific disciplines may exhibit different patterns of vaccine willingness compared to those from non-health-related fields.

Though our data suggest a possible relationship between education level and the ability to interpret complex health information, this interpretation remains exploratory. Further investigation is warranted to clarify the mechanisms behind this association and to inform the design of interventions aimed at promoting vaccine acceptance.

## Conclusions

This study found a significant positive relationship between education level and COVID-19 vaccine willingness, importance, and understanding across our multinational sample. Individuals with higher reported educational attainment were more willing to receive the vaccine and demonstrated greater recognition of its importance and rationale. These findings emphasize the potential role of education and health literacy in improving vaccine acceptance.

Targeted efforts to enhance health education and improve access to accurate vaccine information among populations with lower educational attainment may reduce vaccine hesitancy. Future research should explore how educational interventions can strengthen critical evaluation of vaccine-related information and promote informed health decisions.

## Appendices

COVID-19 vaccination survey

Gender:

o   Female

o   Male

o   Other

Age:

o   18-24

o   25-34

o   35-44

o   45-54

o   55-64

o   65+

Level of education attained:

o   Some high school

o   Completed high school

o   Some college or university

o   Completed college or university

Have you received at least one dose of the COVID-19 vaccine?

o   Yes

o   No

In what country do you live?

_______________________________________

Rate the following statement: 

"Receiving the COVID-19 vaccine is important for protecting myself and my community."

o   Strongly agree

o   Agree

o   Neither agree nor disagree

o   Disagree

o   Strongly disagree

Rate the following statement: 

"I understand how vaccines help reduce the spread of disease in communities and help establish herd immunity."

o   Strongly agree

o   Agree

o   Neither agree nor disagree

o   Disagree

o   Strongly disagree

Rate your willingness to receive the COVID-19 vaccine:

o   Very willing

o   Somewhat willing

o   Not so willing

o   Not at all willing

o   Not at all willing and did not get vaccinated
